# A scoping review of studies on the health impact of electronic nicotine delivery systems

**DOI:** 10.1007/s11739-021-02835-4

**Published:** 2021-10-12

**Authors:** Cother Hajat, Emma Stein, Saran Shantikumar, Raymond Niaura, Pietro Ferrara, Riccardo Polosa

**Affiliations:** 1 Public Health Institute, UAE University, Al Ain, United Arab Emirates; 2 Atlanta, USA; 3 Warwick Medical School, University of Warwick, Coventry, UK; 4 School of Global Public Health, New York University, New York, USA; 5 Centre for Public Health Research, University of Milan - Bicocca, Monza, Italy; 6 Center of Excellence for the Acceleration of HArm Reduction (CoEHAR), University of Catania, Catania, Italy; 7 Department of Clinical and Experimental Medicine, University of Catania, Catania, Italy

**Keywords:** Tobacco harm reduction, E-cigarettes, ENDS, Smoking, Tobacco, Cardiovascular disease, Cancer, Mortality, Respiratory disease, Mental health

## Abstract

We conducted a scoping review of studies on health outcomes from electronic nicotine delivery systems (ENDS). The objective was to identify, narratively synthesize, assess the strength and quality of evidence and critically appraise studies that have reported disease end points associated with the use of ENDS. We included published literature on the health impact of ENDS from 01/01/2015 until 01/02/2020 following the PRISMA guidelines using PubMed, Embase, Scopus and Google Scholar. The database search identified 755 studies, and other sources 265; 37 studies met final eligibility criteria. Levels of evidence included 24(65%) cross-sectional, one (2.7%) case–control and six (16%) case studies, four (11%) cohort studies, one (2.7%) randomized controlled trial (RCT) and one (2.7%) meta-analysis; 27(73%) studies reported only on harms, eight (22%) reported on benefits, two (2%) on benefits and harms. Quality ratings were poor in 20 (54%), fair in 9(24%) and good in 8(22%) of studies. In our review, ENDS was not shown to be causative for harmful cardiovascular disease (CVD) outcomes and shown to be beneficial for hypertensive patients. Switching from cigarettes to e-cigarettes resulted in reduced exacerbations of chronic obstructive pulmonary disease (COPD), with no evidence of long-term deterioration in lung function. Mental Health, cancer and mortality were not adequately studied to form any consensus. Our review has not demonstrated ENDS to be causative of harmful CVD outcomes; furthermore switching from cigarettes to e-cigarettes was associated with improved hypertensive control and reduced exacerbations of COPD, with no evidence of increased asthma risk or long-term respiratory harm. Mental health, cancer and mortality outcomes have not been adequately studied to form a conclusion. Overall, the findings of our review did not provide evidence to counter the consensus held by many that ENDS use is safer than the risks posed from smoking cigarettes.

## Introduction

Smoking is the leading preventable cause of illness and premature death and one of the top causes of health inequalities, responsible for over eight million deaths a year globally [1].

The availability of tobacco harm reduction (THR) products has dramatically accelerated the reduction in the smoking prevalence rate [2]. Electronic nicotine delivery devices (ENDS), such as electronic cigarettes and vapes, are amongst the most effective smoking cessation methods [3, 4] due to a combination of successful quit rates [5] and their greater reach and accessibility compared with other smoking cessation methods [2].

The prevalence of the use of ENDS is highest in the UK (6%) and the US (4–6%) compared with 1% of the rest of Europe [2]. The vast majority of regular ENDS users are previous or current smokers: in the UK over 99% of adult users and over 99.5% of adolescent users are former smokers [2]; and in the US, 98.7% of adults aged 45 years or older and 60% of adults aged 18–24 years were former smokers [6].

To determine the net health impact of ENDs, the benefits from quitting smoking must be weighed against any harms (or benefits) from the use of ENDS. To date, there has been no clear consensus on the safety profile of ENDS and safety concerns have resulted in varying regulations and bans on their sale and use globally.

Studies investigating the safety profile of ENDS include chemical, toxicological and clinical studies. Chemical studies cannot provide novel safety information as they rely on theoretical models and pre-determined safety levels which are unavailable. Toxicological studies are mostly cytotoxicity studies on established cell lines which cannot be accurately extrapolated to the in-vivo situation as there are too many assumptions and unknowns from the behaviour of cell lines and ENDS product variables such as heat, concentrations and amount of product delivery. Despite this, policy decisions on ENDS and THR products are made using animal, in vitro and in silico studies which may not translate to health outcomes in real-world settings.

A widely used estimate for health risk by Public Health England is that e-cigarettes pose less than 5% risk of conventional cigarettes [7]. More recently the US National Academies of Sciences, Engineering and Medicine (NASEM) consensus is that e-cigarettes are “likely to be far less harmful” than combustible cigarettes [8]. There have been no meta-analyses or systematic reviews to quantify the health risk posed by ENDS to date.

The objective of our scoping review was to identify, narratively synthesize, assess the strength and quality of evidence and critically appraise studies that have reported disease end points associated with the use of ENDS.

## Methods

We systematically reviewed published literature on the health impact of ENDS products. We included all electronic nicotine delivery devices (not including heat-not-burn products). The study followed PRISMA guidelines for reporting of systematic reviews [9]. We included health outcomes of new onset or control of disease end-points. We did not include other health outcomes such as short-term physiological changes which do not necessarily manifest as disease, quality of life, studies of emissions only, or those arising from departure from intended use of ENDS devices such as explosions, or use of ENDS devices to vapourise alternative products.

### Search strategy and eligibility criteria

A literature search was conducted between 1st October 2019 and 26th February 2020 using the databases PubMed, Embase, Scopus and Google Scholar using medical subject headings. There were two domains for the search, one for use of ENDS and related products and one for health outcomes, specifically cardiovascular disease (CVD), cancer, respiratory, mortality and ‘other’ health outcomes.

Search terms included (“Electronic cigarette” OR “Electronic nicotine delivery system” OR “E-cigarette” OR “Vaping” OR “Vapor” OR “Reduced risk tobacco product” OR “Non cigarette tobacco” OR “Nicotine aerosol” OR “E-cigarette aerosol”) AND (“health outcome” OR “Morbidity” OR “Mortality” OR “Cancer” OR “Cardiovascular disease” OR “Chronic obstruct pulmonary disease” OR “COPD” OR “CVD” OR “Acute myocardial infarction” OR “Stroke” OR “Cardiovascular” OR “Cerebrovascular” OR “Health effects” OR “Adverse” OR “effects” OR “Respiratory”).

Search results were filtered to include only English language, human studies and published from 01/01/2015 until 01/02/2020. Because most ENDS use has fallen within this period and ENDS products have evolved considerably since 2015. The references of relevant reviews were manually searched for additional eligible citations.

The titles, abstracts and full texts of the search results were sequentially screened by two reviewers independently for inclusion using the eligibility criteria below, with disagreements resolved via blind review by a third reviewer.

**Fig. 1:**
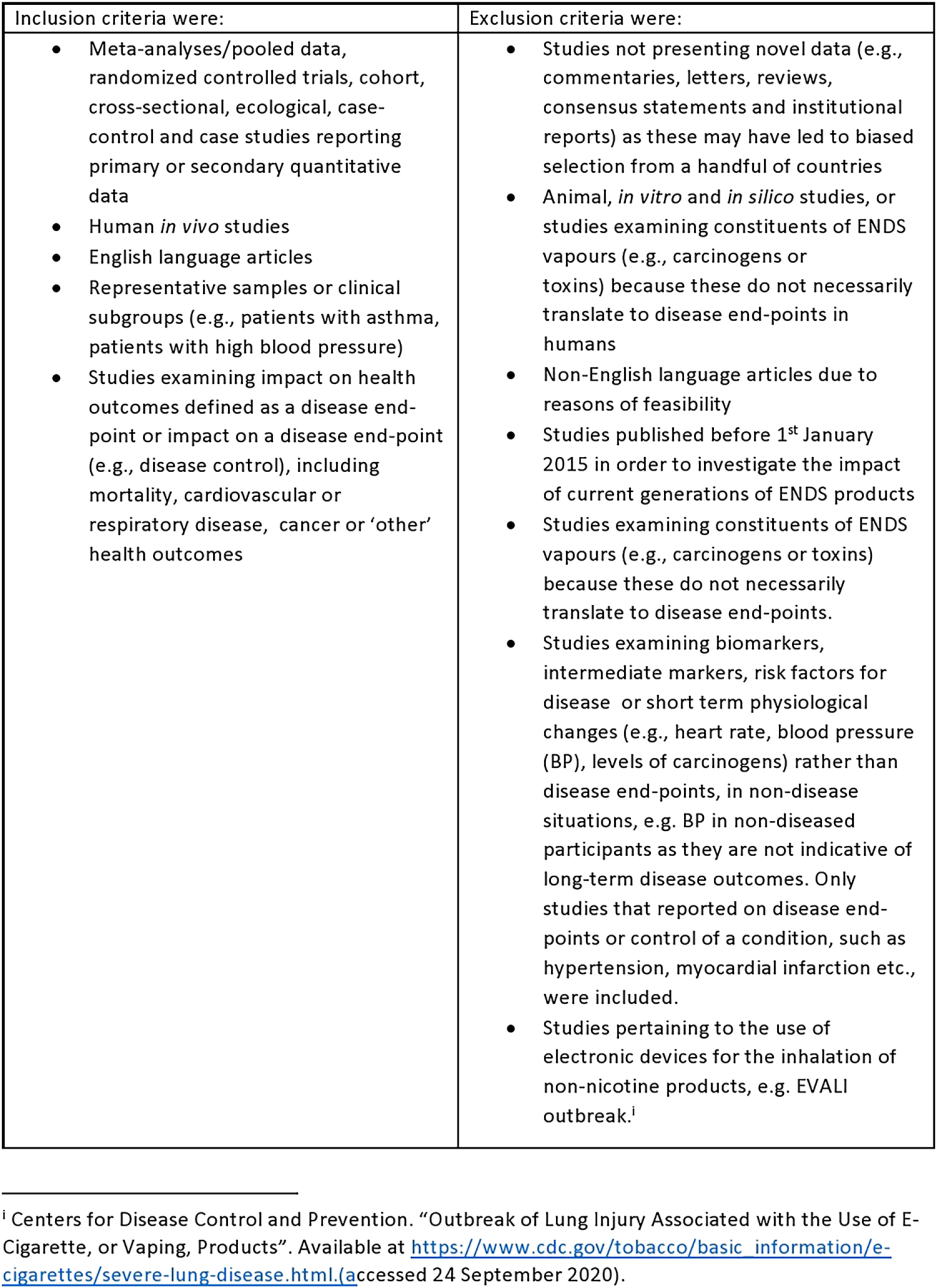
Inclusion and exclusion criteria

Figure 1 shows the inclusion and exclusion criteria used.

### Data extraction and quality assessment

For included studies, data were extracted including author, year, country, aim, study design, sample size, participants and relevant findings such as effect sizes and nature of impact on health outcomes. A level of evidence category was assigned using the Oxford Centre for Evidence-Based Medicine framework[10] and a similar approach used to categorise methodological quality as “good”, “fair” or “poor” utilizing the National Institutes for Health (NIH) Quality Assessment Tools [11]. The NIH quality assessment tools include features to assess the risk of bias, such as selection and reporting bias, with a “good” rating reflecting a low risk of bias, and a “poor” rating suggesting a high risk of bias. A review of common sources of bias encountered in the included literature is given in the discussion. Data extraction and synthesis were formed by two reviewers independently with blind assessment by a third reviewer for cases with rater disagreement. Findings of all studies were independently reviewed, coded and compared between studies to identify relationships and themes.

## Results

**Fig. 2:**
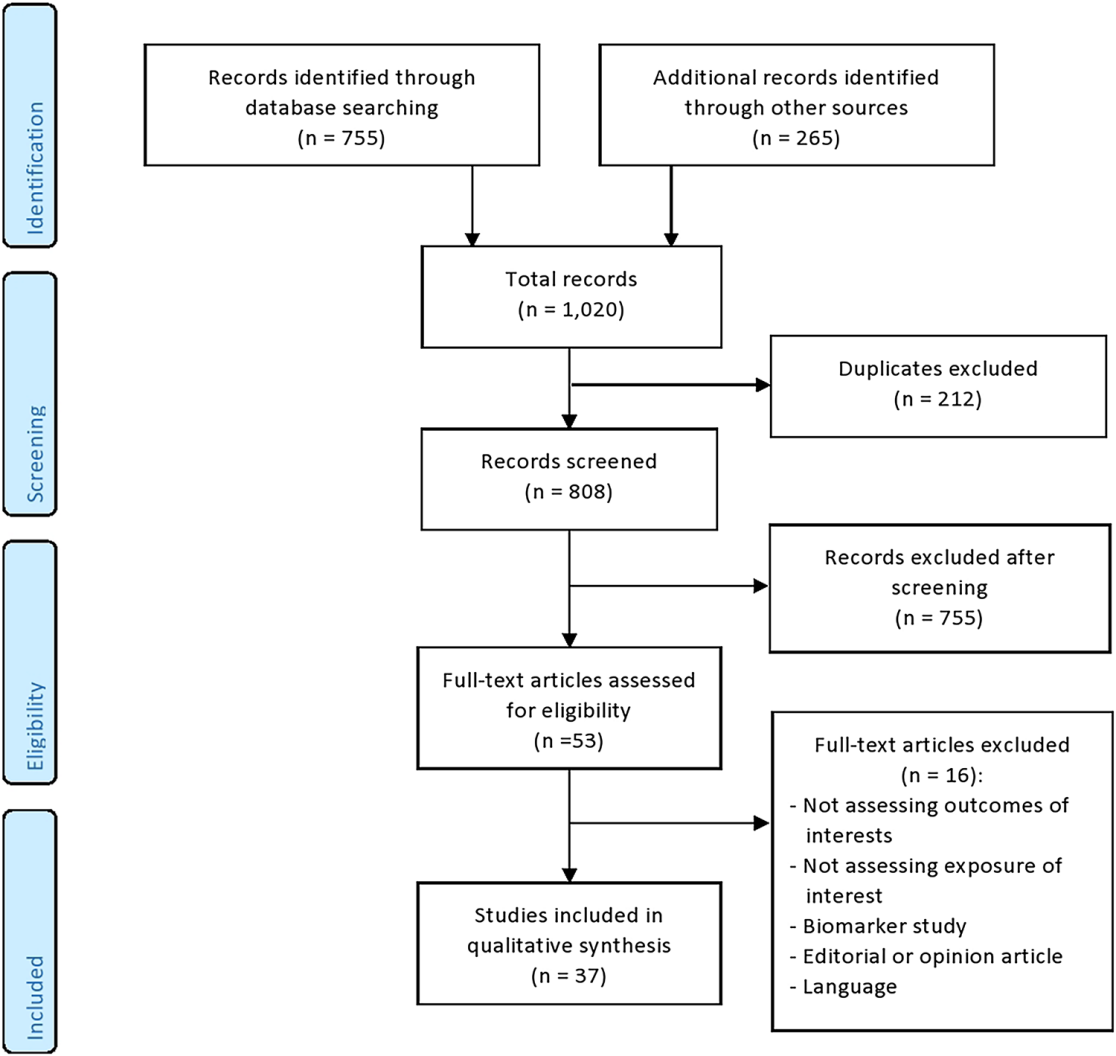
PRISMA flow chart of included studies and selection process

Thirty-seven studies were included in the review. Reasons for excluding studies are shown in Fig. 2.

Table 1 shows that the types of studies were: cross-sectional (24 studies, 65%), case–control (1, 2.7%) and case studies (6, 16%), cohort (1, 11%), randomized controlled trial (RCT; 1, 2.7%), MA/pooled study (1, 2.7%). Each of the 37 studies only examined one of the listed health outcomes. There were 17 studies on respiratory disease [12–14, 22–35, 52] followed by CVD (6) [16–21] mental health (7), [37–42, 54] and one each on oral health[43], two on cancer [15, 36], self-reported chronic health conditions [44], tonsillitis [35] and nickel contact allergy [46].

**Table 1:** Number of studies by health outcome and study design

	Cancer	Respiratory	Cardiovascular	Mental health	Oral health	Mortality	Other	Total
Ma/pooled data		1					1	1
Rct			1					1
Cohort		2		2				4
Cross-sectional	1	11	5	5	1		1	24
Case–control			1					1
Ecological								0
Case report	1	3	1				1	6
All articles	2	17	7	7	1	0	3	37

Table 2 summarises that 27 (73%) of the studies reported only on harms, eight (22%) on benefits, two (2%) on both harm and benefits. Although few in number, studies investigating benefits were of higher levels of evidence with one RCT, three cohort (two examining both harms and benefits), four cross-sectional, one case–control and one case study.

**Table 2:** Study design by reporting of harms and benefits

	Harms	Benefits	Both/neutral
Meta-analysis/pooled data	1		
Rct		1	
Cohort	1	1	2
Cross-sectional	20	4	
Ecological			
Case-control		1	
Case report	5	1	
All articles	27	8	2

Table 3 summarizes the quality ratings assigned to studies by health outcome. Raters one and two agreed on 32 out of 37 (94%) assessments of quality and level of evidence. “Poor” quality studies made up 20 (54%), “fair” made up nine (24%) and “good” made up eight (22%), with reasons including insufficient follow-up, inability to determine temporality and reverse causation, inadequate accounting for confounders and poor definitions of exposures and outcomes.

**Table 3:** Quality ratings assigned to studies by health outcome

Health outcome	Good	Fair	Poor
ENDS			
Mortality	0	0	0
Cvd	2 (29%)	2 (29%)	3 (43%)
Respiratory	3 (18%)	3 (18%)	11 (65%)
Cancer	0 (0%)	2 (100%)	0 (0%)
Oral health	0 (0%)	0 (0%)	1 (100%)
Mental health	2 (29%)	1 (14%)	4 (57%)
Other	1 (33%)	1 (33%)	1 (33%)
Total	8 (22%)	9 (24%)	20 (54%)

Characteristics of included studies including study design, key outcomes, level of evidence and quality ratings are detailed in Table 4 and in further detail in Table 5 (appendix).

**Table 4:** Health outcomes of included studies

Study	Subjects	Outcome	Impact on health outcome	Evidence	Quality
Farsalinos [17]	145	SBP and HR	From 141 to 132 mmHg, *p* < 0.001	1B	Good
Alzahrani [20]	6904	MI	OR = 1.79; 95% CI 1.20–2.66	2C	Poor
Farsalinos [17]	33,028, 26,742	MI and CHD	OR = 1.35; 95% CI 0.80–2.27	2C	Fair
Osei [18]	449,092	CVD	OR = 1.04; 95% CI 0.63–1.72	2C	Poor
Parekh	161,529	Stroke	OR = 0.69, 95% CI 0.34, 1.42	2C	Fair
Polosa [16]	89	SBP and DBP	40–130 mmHg; *p* < 0.001; 86–80 mmHg; *p* = 0.006	3B	Good
Bowler [22]	4596	COPD respiratory symptoms	*p* = 0.01	2A	Good
Polosa [23]	44	COPD exacerbations	*p* = 0.019; *p* = 0.001	2B	Good
Lappas [33]	54	Impulse oscillometry impedance	*p* = 0.022	2B	Poor
Polosa [24]	48	COPD exacerbations	Mean 2.3 at baseline to 1.8; *p* = 0.002	2C	Good
Miler [35]	914	Respiratory infections	66% (95% CI 62.9–69.0)	2C	Poor
Bhatta [53]	705,159	Chronic bronchitis, emphysema, COPD	OR = 1.75, 95% CI 1.25–2.45	2C	Poor
Cho [30]	35,904	Asthma	OR = 2.74; 95% CI 1.30–5.78	2C	Poor
Choi [31]	36,085	Asthma attacks	OR = 1.78, 95% CI 1.20–2.64	2C	Poor
Kim [32]	216,056	Asthma	OR = 1.13; 95% CI 1.01–1.26	2C	Poor
Osei [25]	5454	COPD	OR = 2.94, 95% CI 1.73–4.99	2C	Poor
Perez [26]	32,320	COPD	OR = 1.43, 95% CI 1.12–1.85	2C	Poor
Schweitzer [34]	6082	Asthma	OR = 1.48; 95% CI 1.24–1.78	2C	Poor
Wills [27]	8087	Asthma or COPD	OR = 1.33, 95% CI 1.00–1.77, *p* < 0.05	2C	Fair
Xie [28]	887,182	COPD	OR = 1.47; 95% CI 1.01, 2.12	2C	Fair
Sommerfeld [14]	1	Dyspnea, cough, and pleuritic chest pain after e-cigarette use	Case study of single EC user developing sensitivity pneumonitis	4	Fair
Khan [13]	1	Organising pneumonia	Single case study of organizing pneumonia, exclusion of other drug use and comorbidities not mentioned	4	Poor
Carter [12]	1	Vesicular Bronchial Injury	Case study showed vesicular bronchial injury in an EC user. Patient had CVD and other comorbidities and was a former smoker	4	Poor
Franco [36]	65	Oral mucosa pre-cancerous cells	*p* = 0.001; *p* = 0.004	2C	Fair
Nguyen [15]	2	Oral carcinoma	Two cases of oral carcinoma associated with 13-year use of EC. Description of other risks not detailed	4	Fair
Akinkugbe [43]	13,650	Dental problems	OR = 1.11; 95% CI 0.79–1.55	2C	Poor
Bandiera [37]	5445	Depressive symptoms	*β* = 0.05; *p* < 0.01	2B	Good
Lechner [38]	347	Depressive symptoms	*b* = 1.272, SE = 0.513, *p* = 0.01	2B	Good
Dahal [39]	52,956	Depressive symptoms	OR = 2.46; 95% CI 1.82–3.33	2C	Poor
Chadi [41]	26,821	Depressive symptoms, suicidal ideation	OR = 1.23; 95% CI 1.03–1.47	2C	Poor
Grant [54]	3572	Mental health issues	*p* = 0.052	2C	Fair
King [40]	2370	Depression	OR = 1.04; 95% CI 1.01–1.08	2C	Fair
Pham [42]	53,050	Mood disorders	OR = 1.9; 95% CI 1.2–3.0	2C	Poor
Lanza [44]	452	BMI	*β* = 1.48, OR = 4.40, *p* < 0.05	2C	Poor
Miler [45]	1	Exacerbations of tonsillitis	After 8 months of vaping, the patient reported absence of exacerbations of tonsillitis, and marked improvement in Tonsillitis. The study did not mention any other comorbidities or exhaustively account for all confounders	4	Fair
Maridet [46]	1	Clinically-determined erythematous, scaly dermatitis	Patient diagnosed with nickel contact dermatitis associated with the use of an EC	4	Good

**Table 5:** Description of studies, level of evidence and quality for health outcomes from ENDS

Benefit or harm	Reference	Country	Study design and number of subjects, country	Key outcomes assessed	Impact on health outcome	Level of Evidence	Quality Rating
Cardiovascular							
Benefit	Farsalinos et al. effect of continuous smoking reduction and abstinence on blood pressure and heart rate in smokers switching to electronic cigarettes	Italy	Prospective, double-blind, controlled, three-arm RCT on 145 hypertensive smokers switching to EC	Clinic measured SBP and HR	Reduced SBP (from 141 to 132 mmHg, *p* < 0.001) in hypertensives switching to EC at 12 months; those continuing to smoke had no reduction	1B	Good
Harm	Alzahrani, et al. association between electronic cigarette use and myocardial infarction	United States	Cross-sectional survey on 6904 subjects	Self-reported MI	Self-reported daily EC users more likely to report MI compared with never EC users (OR = 1.79; 95% CI 1.20–2.66). Compared to never users of e-cigarettes and cigarettes, daily dual users of e-cigarettes and cigarettes were more likely to have an MI (OR = 4.62)	2C	Poor
Harm	Farsalinos et al. is e-cigarette use associated with coronary heart disease and myocardial infarction?	Italy	Cross-sectional survey in 2016 (*n* = 33,028) and 2017 (*n* = 26,742)	Self-reported MI and CHD	Self-reported daily EC use not associated with MI (OR = 1.35; 95% CI 0.80–2.27) compared with never EC use after accounting for dual use and former smoking; no association between EC use and CHD compared with never EC use (OR = 1.31; 95% CI 0.79–2.17)	2C	Fair
Harm	Osei, et al. association between e-cigarette use and cardiovascular disease among never and current combustible-cigarette smokers	United States	Cross-sectional survey on 449,092 participants	CVD (defined as Self-reported CHD, MI, stroke)	Self-reported EC using never-smokers had no increased CVD (OR = 1.04; 95% CI 0.63–1.72) or premature CVD (OR = 1.01; 95% CI 0.56–1.83) compared with never EC users; EC using former-smokers were more likely to report CVD (OR = 1.36; 95% CI 1.18–1.56) and premature CVD (OR = 1.45; 95% CI 1.20–1.74) compared with never EC users. Dual smoking and EC use was associated with higher CVD (OR = 1.36; 95% CI 1.18–1.56)	2C	Poor
Harm	Parekh, et al. risk of stroke with e-cigarette and combustible cigarette use in young adults	United States	Cross-sectional survey on 161,529 participants aged 18−44 years	Self-reported stroke	Self-reported EC using never-smokers had no higher risk of stroke (OR = 0.69, 95% CI 0.34, 1.42) compared with nonsmokers; risk of stroke was lower for EC users compared with current exclusive smokers (OR = 0.43, 95% CI 0.20, 0.93). Current EC using former smokers had increased odds of stroke (OR = 2.54; 95% CI 1.16–5.56) compared with never -smokers	2C	Fair
Benefit	Polosa, et al. blood pressure control in smokers with arterial hypertension who switched to electronic cigarettes	Italy	Observational study of 89 hypertensive smokers who quit or reduced tobacco consumption by switching to EC	Office SBP and DBP	A significant reduction in median SBP (from 140 to 130 mmHg; *p* < 0.001) and DBP (from 86–80 mmHg; *p* = 0.006) at 12-month follow-up in the exclusive EC group. No change in SBP or DBP seen in reduced cigarette consumption dual users at 12 months	3B	Good
Respiratory							
Harm	Bowler et al. Electronic cigarette use in US adults at risk for or with COPD: analysis from two observational cohorts	United States	Pooled results from two cohort studies in 4,596 current or former smokers Aged 45–80 with, or at risk of, COPD	COPD respiratory symptoms or disease progression (GOLD criteria used to assess COPD spirometric severity)	Self-reported ever use of EC associated with 8% (± 2%) increased prevalence of chronic bronchitis and (in 1 cohort) COPD exacerbations compared with never EC users (*p* = 0.01); after 5 years, no increase in progression of lung disease or decline in lung function (in one cohort). Adjusted for but not excluding current smokers	2A	Good
Benefit	Polosa et al. health effects in COPD smokers who switch to electronic cigarettes: a retrospective-prospective 3-year follow-up	Italy	Prospective cohort study of 44 COPD smokers switching to EC use	COPD exacerbations, post-bronchodilator lung function, CAT scores, 6-min walking distance	Improvements in COPD exacerbation rates (*p* = 0.004), CAT scores (*p* = 0.019) and 6-min walk distance (*p* = 0.001) in EC users compared with continued smokers after 36 months	2B	Good
Harm	Lappas et al. short‐term respiratory effects of e‐cigarettes in healthy individuals and smokers with asthma	Greece	Cohort study of 54 dual smokers (EC and smoking), 27 (50%) with mild asthma (MA), 27 (50%) no asthma, underwent a control session (no liquid, no resistor coil inside e-cigarette cartridge) and an experimental session of EC using standardized puffing settings	Impulse oscillometry impedance (Z), resistance (R), reactance (X) and fractional exhaled nitric oxide (FeNO) were measured before and 0, 15 and 30 min after control and experimental sessions	MA group exhibited higher baseline values and more prominent effect after EC use using standardized puffing sessions vs. healthy participants after EC use for respiratory system total impedance at 5 Hz (*p* = 0.022), respiratory system resistance at 5 Hz (*p* = 0.010) and respiratory system resistance at 10 Hz (*p* = 0.013). Fractional exhaled nitric oxide decreased significantly in both groups (*p* < 0.001)	2B	Poor
Benefit	Polosa et al. evidence for harm reduction in COPD smokers who switch to electronic cigarettes	Italy	Retrospective chart review with 12 and 24 month follow-up on 48 heavy smokers with COPD invited to switch to e-cigarettes	Verified COPD exacerbations in previous 12 months	Reduction in annual COPD exacerbations for heavy smokers with COPD switching to EC (mean 2.3 at baseline to 1.8; *p* = 0.002) at 12 months and to 1.4; *p* < 0.001) at 24 months,: no change in COPD exacerbations for those not switching	2C	Good
Benefit	Miler et al. changes in the frequency of airway infections in smokers who switched to vaping: results of an online survey	Germany	Cross-sectional survey of 914 smokers who switched to vaping for at least 2 months	Self-reported respiratory infections (e.g., common cold)	Among those who switched to EC, 66% (95% CI 62.9–69.0) reported improvement in respiratory infections, 29% reported no change, 5% reported worsening	2C	Poor
Harm	Bhatta et al. association of e-cigarette use with respiratory disease among adults: a longitudinal analysis	United States	Cross-sectional survey of 705,159 participants	Self-reported chronic bronchitis, emphysema, COPD	Among never smokers, current self-reported EC use associated with chronic bronchitis, emphysema and COPD compared with never EC users (OR = 1.75, 95% CI 1.25–2.45); daily EC use had higher odds (OR = 2.64, 95% CI 1.43, 4.89)	2C	Poor
Harm	Cho et al. association between electronic cigarette use and asthma among high school students in South Korea	South Korea	Cross-sectional survey of 35,904 high school students	Self-reported asthma diagnosis	Among self-reported never smokers, current EC use associated with asthma (OR = 2.74; 95% CI 1.30–5.78) compared with never EC users	2C	Poor
Harm	Choi, Bernat e-cigarette use among Florida youth with and without asthma	United States	Cross-sectional survey of 36,085 high school students	Self-reported asthma and asthma attack	Among those with asthma, self-reported past 30-day EC use (any quantity) associated with asthma attacks (OR = 1.78, 95% CI 1.20–2.64) in the past 12 months compared with non EC users in past 30 days (adjusted for days smoked cigarettes in the past 30 days but smokers not excluded)	2C	Poor
Harm	Kim et al. active, passive, and electronic cigarette smoking is associated with asthma in adolescents	South Korea	Cross-sectional survey of 216,056 adolescents aged 12–18 years	Self-reported asthma	Self-reported EC use group associated with higher prevalence of asthma (OR = 1.13; 95% CI 1.01–1.26) compared with never EC users (adjusting for active, passive cigarette use); greater use of e-cigarettes associated with asthma, 1–5 days/month (OR = 1.39; 95% CI 1.19–1.61), 6–19 days/month (OR = 1.31; 95% CI 1.08–1.61) and > 20 days/month (OR = 1.58; 95% CI 1.40–1.78) compared with never EC use	2C	Poor
Harm	Osei et al. association between E-cigarette use and chronic obstructive pulmonary disease by smoking status: behavioral risk factor surveillance system 2016 and 2017	United States	Cross-sectional survey on 5454 participants	Self-reported COPD diagnosis	Self-reported non-current smokers using EC associated with a COPD diagnosis (OR = 2.94, 95% CI 1.73–4.99) compared with non-EC use. Compared with never smokers who never used EC, dual users (smoking and EC) had the highest odds of COPD (OR = 6.89, 95% CI = 6.29, 7.55) Former smoking was not excluded or accounted for	2C	Poor
Harm	Perez et al. adult e-cigarettes use associated with a self-reported diagnosis of COPD	United States	Cross-sectional survey of 32,320 adults and adolescents aged 12–17 years	Self-reported COPD diagnosis	Self-reported EC users had greater odds of COPD than non- EC users (OR = 1.43, 95% CI 1.12–1.85) in adults and children combined	2C	Poor
Harm	Schweitzer et al. e-cigarette use and asthma in a multiethnic sample of adolescents	United States	Cross-sectional survey of 6,082 adolescents	Self-reported asthma diagnosis	Current self-reported EC use associated with asthma (OR = 1.48; 95% CI 1.24–1.78) and with previous asthma (OR = 1.20; 95% CI 1.00–1.44) compared with never EC use, (controlling for but not excluding current cigarette smoking, or former smoking)	2C	Poor
Harm	Wills et al. e-cigarette use and respiratory disorder in an adult sample	United States	Cross-sectional random-dial telephone survey on 8087 adults	Self-reported asthma or COPD diagnosis	Self-reported ever EC use associated with asthma in current non-smokers (OR = 1.33, 95% CI 1.00–1.77, *p* < 0.05) but not in smokers (OR = 0.92, 95% CI 0.73–1.15, EC use was not associated with COPD in current non-smokers (OR = 2.98, 95% CI 1.51–5.88, *p* < 0.01) or in current smokers (OR = 1.29, 95% CI 0.94−1.77). There was no significant difference in risk of asthma among dual users compared with sole EC users (OR = 1.00; 95% CI 0.73–1.35) or smokers (OR = 0.99; 95% CI 0.80–1.22). There was increased risk of COPD in smokers (OR = 2.98; 95% CI 2.34–3.78), EC users (OR = 2.58; 95% CI 1.36–4.89) and dual users (OR = 3.92; 95% CI 2.82–5.44) compared with never smokers who never used EC. Ever EC use included any quantity ever used	2C	Fair
Harm	Xie et al. use of electronic cigarettes and self-reported chronic obstructive pulmonary disease diagnosis in adults	United States	Cross-sectional survey of 887,182 participants	Self-reported COPD diagnosis	Self-reported current vapers who never smoked more likely to self-report COPD (OR = 1.47; 95% CI 1.01, 2.12) compared with never smokers (smoked less than 100 cigarettes, not currently vaping)	2C	Fair
Harm	Sommerfeld et al. hypersensitivity pneumonitis and acute respiratory distress syndrome from e-cigarette use	United States	Case study, 18-year-old woman with dyspnea, cough, and pleuritic chest pain after e-cigarette use	Hypersensitivity Pneumonitis and acute respiratory distress syndrome	Case study of single EC user developing sensitivity pneumonitis. Did not report on comorbidities or smoking	4	Fair
Harm	Khan et al. organizing pneumonia related to electronic cigarette use: a case report and review of literature	United States	Case study, 40-year-old female patient	Organising pneumonia	Single case study of organizing pneumonia, exclusion of other drug use and comorbidities not mentioned	4	Poor
Harm	Carter et al. Life-threatening vesicular bronchial injury requiring veno-venous extracorporeal membrane oxygenation rescue in an electronic nicotine delivery system user	United States	Case study, 35-year-old female presented to emergency department with chest pain and dyspnea	Vesicular Bronchial Injury	Case study showed vesicular bronchial injury in an EC user. Patient had CVD and other comorbidities and was a former smoker	4	Poor
Cancer							
Benefit	Franco et al. electronic cigarette: role in the primary prevention of oral cavity cancer	Italy	Cross-sectional survey on 65 previous smokers (from outpatient center), e-cigarette smokers (from monthly prevention campaigns), and nonsmokers (from university medical and paramedical staff)	Total number of oral mucosa pre-cancerous (micronucleated) cells from cytologic examination	Self-reported EC users had lower micronuclei compared with smokers based on micronucleated cells/1000 cells (*p* = 0.001) and micronuclei/1000 cells (*p* = 0.004)	2C	Fair
Harm	Nguyen et al. oral carcinoma associated with chronic use of electronic cigarettes	United States, Vietnam	Case study of 2 subjects	Oral carcinoma	Two cases of oral carcinoma associated with 13-year use of EC. Description of other risks not detailed eg smoking	4	Fair
Oral health							
Harm	Akinkugbe et al. cigarettes, e-cigarettes, and adolescents’ oral health: findings from the population assessment of tobacco and health (PATH) study	United States	Cross-sectional study on 13,650 adolescents aged 12–17 years	Dental problems (cavities, gum disease or dental stains)	No relationship between self-reported EC use and self-reported dental problems, including among current eEC users (OR = 1.11; 95% CI 0.79–1.55) or ever users (OR = 1.12 95% CI 0.90–1.38) compared with never cigarette or EC users	2C	Poor
Mental health							
Harms and benefits	Bandiera et al. depressive symptoms predict current e-cigarette use among college students in Texas	United States	Cohort study of 5445 college students (18–29-year-olds) with 6-month and 1-year follow-ups	Self-reported depressive symptoms	Correlation between depressive symptoms and self-reported EC use was significant at baseline (*β* = 0.05; *p* < 0.01), however, EC use did not predict higher depressive symptoms at 6-months or 1-year follow-up	2B	Good
Neutral	Lechner et al. bi-directional associations of electronic and combustible cigarette use onset patterns with depressive symptoms in adolescents	United States	Cohort study of 347 adolescents assessed at baseline, 6- and 12-month follow-up	Self-reported depressive symptoms	Self-reported EC use over previous 12-months associated with greater rate of increase in depressive symptoms over time (b = 1.272, SE = 0.513, *p* = 0.01) compared with never EC use. Higher frequency of EC use was associated with higher depressive symptoms at 12 months among sustained users (*B* = 1.611, *p* = 0.04)	2B	Good
Benefit	Dahal et al. smoking cessation and improvement in mental health outcomes: Do people who quit smoking by switching to electronic cigarettes experience improvement in mental health?	Canada	Cross-sectional survey on 52,956 participants	Self-reported depressive symptoms	Self-reported EC use (any quantity) who were never smokers had higher depressive symptoms (≥ 10 on CES-D 10) compared with never EC users (OR = 2.46; 95% CI 1.82–3.33). Former smokers who used ECs had higher depressive symptoms compared with never smokers (OR = 4.19; 95% CI 2.47–7.11). Former smokers who did not use EC had elevated risk of depressive symptoms as well (OR = 1.41 (95% CI 1.19–1.68) compared to never smokers. EC use included any quantity including experimental use	2C	Poor
Harm	Chadi et al. depressive symptoms and suicidality in adolescents using e-cigarettes and marijuana: a secondary data analysis from the youth risk behavior survey	United States	Cross-sectional survey of 26,821 high school students	Self-reported depressive symptoms and suicidal ideation	Self-reported EC use associated with higher odds of suicidal ideation in past 12 months (OR = 1.23; 95% CI 1.03–1.47) and depressive symptoms (OR = 1.37; 95% CI 1.19–1.57) compared with never EC users, adjusted for current smoking (but former and current smokers were not excluded). No use of validated scores to obtain outcomes	2C	Poor
Harm	Grant et al. e-cigarette use (vaping) is associated with illicit drug use, mental health problems, and impulsivity in university students	United States	Cross-sectional survey of 3572 college and graduate school students	Self-reported mental health issues on PHQ9 scale, self-reported diagnosis of ADHD (Y/N), PTSD (PC-PTSD score), gambling disorder (Y/N), anxiety (GAD-7 score), trait impulsivity plus compulsivity, academic impairments	Self-reported EC use associated with mental health issues, including PHQ-9 score ≥ 10 (Cramer’s *V* = 0.044; *p* = 0.052), ADHD (Cramer’s *V* = 0.073; *p* < 0.001)), PTSD (PC-PTSD score ≥ 3;Cramer’s *V* = 0.064; *p* = < 0.002), gambling disorder (Cramer’s *V* = 0.081, *p* < 0.001) and anxiety (GAD-7 > 10; Cramer’s *V* = 0.066; *p* < 0.001). They were also more likely to report low self-esteem (Cramer’s *V* = 0.63; *p* = 0.002), and endorse traits of impulsivity (attentional: cohen’s *d* = 0.421; *p* < 0.001), but not compulsivity (cohen’s *d* = 0.532; *p* = 0.043). Did not control for cigarette use. Participation rate of 38% so sample bias possible. No definition of EC use provided	2C	Fair
Harm	King et al. tobacco product use and mental health status among young adults	United States	Cross-sectional survey of 2370 college students	Self-reported depression (higher score, greater depression), stress (higher score, greater perceived stress), mental health diagnosis	Self-reported EC use associated with higher depression score (OR = 1.04; 95% CI 1.01–1.08) compared with never EC use, controlling for 30-day cigarette use. EC use was associated with higher stress score (OR = 1.03 95% CI 1.00–1.05) compared with never EC use, controlling for 30-day cigarette use. Dual use but not former smoking was accounted for	2C	Fair
Harm	Pham et al. electronic cigarette use and mental health: a Canadian population-based study	Canada	Cross-sectional survey of 53,050 participants	Self-reported depressive symptoms, mood and anxiety, mental health, suicidal thoughts, binge drinking	Among female non-smokers, self-reported EC users had increased mood disorders (OR = 1.9; 95% CI 1.2–3.0) and anxiety disorders (OR = 1.9; 95% CI 1.1–3.2) compared with non- EC users. Female current EC use was associated with mood (OR = 1.9 (95% CI 1.4–2.6) and anxiety (OR = 2.6 (95% CI 1.9–3.6)) disorders compared with non EC use. Among male non-smokers, self-reported EC users had increased mood disorders (OR = 1.6; 95% CI 1.0–2.7) compared with non-EC users. Among male smokers, EC use was not associated with mood disorders (OR = 1.4 (95% CI 0.9–2.3). EC use was defined as any quantity within the last 3 months, including experimental use	2C	Poor
Other							
Harm	Lanza et al. obesity and cigarette smoking: extending the link to e-cigarette/vaping use	United States	Cross-sectional survey (convenience sample) of 452 participants	Self-reported BMI	Obese (BMI ≥ 25 kg/m^2^) participants had higher likelihood of belonging to self-reported Cigarette/EC/Tobacco class compared with the High Substance Use (*β* = 1.48, OR = 4.40, *p* < 0.05) and Risky Alcohol Use (*β* = 1.94, OR = 6.97, *p* < 0.05) classes; higher likelihood of being classified into the cigarette/electronic tobacco class compared to the low substance use class not significant No detail of definitions for EC use	2C	Poor
Benefit	Miler et al. resolution of recurrent tonsillitis in a non-smoker who became a vaper. A case study and new hypothesis	United Kingdom	Case study of a never-smoker who vapes, with a history of recurrent, chronic tonsillitis	Exacerbations of tonsillitis	After 8 months of vaping, the patient reported absence of exacerbations of tonsillitis, and marked improvement in Tonsillitis. The study did not mention any other comorbidities or exhaustively account for all confounders	4	Fair
Harm	Maridet et al. the electronic cigarette: the new source of nickel contact allergy of the twenty-first century?	France	Case study on a 52-year old woman	Clinically-determined erythematous, scaly dermatitis	The patient was diagnosed with nickel contact dermatitis associated with the use of an electronic cigarette. The articles also discussed the literature on nickel content in different brands of ECs	4	Good

### Overall results for health outcomes by category

#### CVD outcomes

Two studies judged to be of good quality, an RCT and observational study, reported reductions in systolic blood pressure (SBP) by 9–10 mmHg, and diastolic BP (DBP) by 6 mmHg in hypertensive patients [16, 17].

Of another four studies, two were rated as ‘fair’ and two as ‘poor’ quality. Two large cross-sectional surveys on approximately 0.5 million [18] and 60,000 [19] subjects found that users of ENDS had no increase in MI, coronary heart disease (CHD), premature CVD or CVD compared with never smokers. However, former smokers who used ENDS did have more CVD (OR 1.4) and premature CVD (OR 1.5) than never smokers in one of the studies [18]. Dual users experienced higher CVD (OR 1.36) compared with those who were current smokers not using ENDS [18]. A further study that did not account for former smokers or dual users, or for temporality and reverse causation, found users of ENDS to have increased risk of myocardial infarction (MR; OR 1.8) [20].

A large cross-sectional study investigating stroke found no excess risk in users of ENDS in never smokers [21]. The use of ENDS in ex-smokers was associated with a higher risk of stroke (OR 2.5) compared with never smokers [16].

#### Respiratory outcomes

Of 17 studies reporting respiratory outcomes, the majority were on chronic obstructive pulmonary disease (COPD) in adults or asthma in adolescents; only three were rated as ‘high’ quality.

The studies on COPD that were judged to be of low risk of bias were a pooled study of two cohorts [22], and an interventional study over 12 months [23], with further follow-up over 3 years [24]. They reported that COPD exacerbations reduced in frequency in heavy smokers switching to e-cigarettes from 2.3 to 1.4 annually [24], and improvements in verified COPD Assessment Test (CAT) score, walking distance and continued reductions in COPD exacerbations after 3 years [24]. The study pooling findings from two cohort studies [22], without excluding current smokers, reported e-cigarette users to have 8% higher prevalence of chronic bronchitis and COPD exacerbations in one of the two included cohort studies. After 5 years of follow-up, no increased progression of lung disease or decline in lung function was seen in e-cigarette users [22]. Current and former smoking was adjusted for but not excluded.

Five cross-sectional studies [24–28] investigated the association between e-cigarette use and COPD. In one study, [26] 85% of the sample were not in the age-risk category (over 55 years) for COPD [29]. One of the cross-sectional studies on a sample of almost 900,000 never-smokers showed an association (OR 1.5) between e-cigarette use and self-reported COPD compared with non-e-cigarette use [28]. Another study that segmented never and current smokers found an association between e-cigarettes and COPD in smokers (OR 1.3) but not in never-smokers (OR 0.9) [27].

Six studies investigated the development or control of asthma [27, 30–34]. An experimental study showed that following e-cigarette use, respiratory system resistance and impedance were impacted up to 30 min afterwards, but fractional exhaled nitric oxide did not differ between asthmatics and non-asthmatics [28]. Five of the six studies were cross-sectional in design and several relied on children and adolescents self-reporting on e-cigarette use and a diagnosis of asthma in schools and other educational facilities. The definitions of e-cigarette users included experimental and one-time use of e-cigarettes in some studies [30–32, 34]. One study [27] reported separately for never smokers and smokers, and found e-cigarette use to be associated with a higher rate of asthma in smokers (OR 1.3) but not in non-smokers (OR 0.9). The remaining studies reported associations between e-cigarette use and asthma, with OR’s ranging between 1.1 and 2.7 [27, 30, 32, 34].

In a cross-sectional study of 914 smokers who switched to e-cigarettes, 66% reported reductions in the frequency of respiratory infections and 6% reported worsening [35]. Single case studies reported on acute hypoxaemic respiratory failure and organizing pneumonia; organizing pneumonia; sensitivity pneumonitis and vesicular bronchial injury, but none specifically excluded other causes such as dual use, former smoking, other drug use or comorbidities.

#### Cancer

A small cross-sectional study demonstrated lower numbers of oral cancerous cells (50%) and cellular changes (33%) in e-cigarette users who were never smokers compared with smokers (*p* = 0.001) [36]. The only other study was a case study on two individuals.

#### Mental health

Seven studies reported on the association between ENDS use and mental health disease [37–42, 54]. Of two cohort studies rated as ‘good’ [37, 38] one found that those with depressive symptoms were more likely to take up e-cigarette use at 6 months (beta-coefficients 0.06, 0.08), but no greater depressive symptoms than non e-cigarette users at 12 months [37]. Another cohort study [38] found a greater increase in depressive symptoms in e-cigarette users after 12 months (beta = 1.27, *p* = 0.01) compared with non e-cigarette users, with a positive dose–response effect.

Four cross-sectional studies [39–42] reported a positive association between e-cigarette use and self-reported depressive symptoms with wide-ranging ORs from 1.03 to 4.2.

A ‘poor’rated cross-sectional study [54] found an association between e-cigarette use and attention-deficit hyperactivity disorder (ADHD; *V* = 0.073; *p* < 0.001), post-traumatic stress disorder (PTSD; *V* = 0.064; *p* =  < 0.002), gambling disorder (*V* = 0.081, *p* < 0.001), anxiety (*V* = 0.066; *p* < 0.001), low self-esteem (*V* = 0.63; *p* = 0.002) and impulsivity traits (cohen’s *d* = 0.421; *p* < 0.001), without controlling for smoking, a participation rate of 38% and not stating a definition for e-cigarette use.

#### Oral health

A ‘poor’ rated cross-sectional study reported no association with self-reported dental health issues in e-cigarette users compared with never smokers [43].

#### Other health outcomes

A ‘poor’ rated cross-sectional study reported an association between e-cigarette use and obesity (OR 4.4, *p* < 0.05) and alcohol abuse (OR 7.0, *p* < 0.05) [44]. There were two single case studies of e-cigarette use being linked to the improvement of recurrent tonsillitis [35, 45] and occurrence of nickel contact allergy [46].

#### Mortality

No studies were found that investigated mortality related to the use of ENDS.

## Discussion

This is one of the first articles to comprehensively and systematically review health outcomes from ENDS use. The 37 studies identified tended to focus on negative health impacts; the benefits of switching from cigarettes to ENDS, which is the usual pattern of use, was an uncommon outcome measure. Evidence of significant harms to health outcomes from ENDS use was lacking from our review, with most studies being unable to rigorously establish causation. In the handful of adequately rigorously designed studies, no causation was established between the use of ENDS and negative health outcomes. There was some evidence of positive health outcomes in those switching from cigarettes to e-cigarettes, for example in COPD and hypertensive patients, but these findings need replication.

### Levels of evidence, quality and study design

There were no studies rated above 2a for level of evidence, i.e. there were no meta-analyses (MAs) or pooling of RCTs. The vast majority of studies (97%) in our review were observational, hence unable to adequately control for confounders and bias, with only one interventional study. The low number of RCTs perhaps reflects the difficulty of conducting interventional THR studies in real-world settings.

Cross-sectional studies were predominant (41%), without accounting for temporality and reverse causation, which is particularly relevant here as the majority of ENDS users are current or former smokers [1, 5, 47]. Furthermore, those with smoking-related medical conditions such as asthma, COPD and CVD are more likely to switch to ENDS to quit smoking [48]. Without accounting for the temporality of the exposure and outcome, as well as former smoking status, many study findings are inadequate for causal inferences.

Conventionally, guidelines and frameworks classify interventional studies such as RCTs as higher levels of evidence than observational ones. However, for lifestyle behaviours such as smoking and use of ENDS, RCTs are not common and the results would not necessarily be generalizable. Observational studies can provide useful information for the investigation of real-world interventions such as ENDS. This issue is not widely acknowledged in guidelines and frameworks used to rate the level of evidence, (e.g., by the NIH Quality Assessment Tools frameworks used in this study) [11].

Included studies were predominantly rated as being of poor quality. Studies that examined benefits to health outcomes had a relatively higher number of fair or good quality studies (75%) compared with those on harms alone (33%).

### Definition of exposure

The definitions used for smoking and ENDS use varied tremendously and most studies relied on self-reported data for these exposures, which is known to underestimate their true prevalence [49]. Studies also asked children and adolescents in educational settings to self-report their use of cigarettes and ENDS, which are usually prohibited [50].

Studies with poor definitions of exposure failed to account for quantity, duration since quitting and duration of ENDS use, dual and former use of cigarettes and ENDS [17, 18, 21, 22, 39, 42], despite evidence that health outcomes from smoking are dose-dependent [51] Studies using data from the Population Assessment of Tobacco Health (PATH) and Behavioral Risk Factor Surveillance System datasets [18, 21, 26, 28, 43, 52] and others, [37, 40] defined respondents who had ever used a cigarette, other tobacco product or ENDS, even experimental use, as former or current users.

Standard definitions exist for smoking and both quantity and duration of smoking impact health outcomes [53]. Similar approaches should be used to quantify ENDS use. A handful of studies accounted for quantity, duration, dual and former use [16, 28, 38].

### Definitions of outcomes

Both exposures and outcomes were self-reported in the majority of studies, and only 14 (38%) of studies utilized verified health outcomes data. Self-reporting of outcomes is known to be unreliable and prone to bias in some situations. Particularly problematic in this review were several studies that asked children and adolescents in educational settings to self-report on asthma and depressive symptoms both of which could have led to subjective and inaccurate responses [30–32, 34, 50, 54, 55].

### Accounting for smoking status

One of the major design flaws was the failure to account for current, former and dual use of cigarettes [56–58] thereby ignoring that the majority of ENDS users do so to quit or cut down on cigarette smoking [56–58].

Several studies compared health risks for ENDS users with those of never smokers without accounting for former smoking in ENDS users. More meaningful comparisons in this regard would be between exclusive ENDS users who were never smokers against non-ENDS users who were never smokers. To quantify the benefit from switching, former smokers who now exclusively used ENDS should be compared with current smokers, accounting also for the duration of switching, duration of smoking and the quantity of cigarettes smoked.

Despite up to 70% of e-cigarette users reporting dual use, [8] studies did not routinely account for dual use when investigating risk from ENDS, thereby attributing health outcomes to ENDS use when they may have resulted from smoking cigarettes.

### Temporality and reverse causation

Of included studies, 41% were cross-sectional and therefore unable to account for temporality and reverse causation, despite the fact that the majority of ENDS users are previous or current smokers [2, 6]. Furthermore, some health outcomes such as COPD and CVD can take up to decades to develop. Cross-sectional studies in current or former smokers cannot be used to establish temporal precedence as was reported in several studies, one of which has since been retracted [59]. Studies reporting on mental health. in particular, failed to account for reverse causation.

### Publication bias

The ratio of studies on harm versus benefits was high with three-quarters of studies reporting only on harms, and less than a quarter reporting on benefits.

There was more frequent reporting of harmful health outcomes rather than neutral or beneficial ones in the abstract and text of the article [28].

The NIH framework has specific areas of critique, one of which is the search for publication bias in meta-analyses. There were no meta-analyses in this study, only one pooled cohort, and therefore, presence of publication bias was not noted.

### Health outcomes

The majority of health outcomes studied were of respiratory (46%), CVD (22%), cancer (5%), oral health (3%) and mental health (19%).

### Mortality

Overall mortality among smokers is three times higher than non-smokers in the US [60, 61], predominantly due to cancer, respiratory disease and CVD [62–64] and quitting before the age of 40 reduces smoking-related deaths by 90% [2, 62].

It is surprising to find that this is not reflected in the focus of research on harms from ENDS, with zero studies identified over the last 5 years. Whilst this may be partly due to the relatively recent availability of ENDS, it would be feasible to study mortality as an outcome in studies of high-risk groups such as CVD patients.

### Cardiovascular disease

An extensive body of evidence shows that smoking tobacco is causally related to almost all major forms of CVD including accelerated atherosclerosis, acute MI, CHD, stroke, peripheral arterial disease (PAD), aortic aneurysm and sudden death [65, 66] and the benefits of quitting smoking on reduced risk for CHD and CVD mortality have been well documented [67–71].

We had expected to see more studies on the impact of switching from cigarette smoking to ENDS on CVD outcomes. The recent availability of ENDS may be partly responsible although other diseases such as COPD have been reported within the same timelines.

Our review found that ENDS product use has not been shown to be causative for harmful CVD outcomes and, indeed, has been shown to be beneficial for hypertensive patients. The finding from one large US cross-sectional study that dual e-cigarette and combustible cigarette users had higher CVD than smokers who had never used e-cigarettes falls outside of the overall findings, although this could be due to individuals with previous CVD being more likely to start using e-cigarettes as a means to reduce tobacco use. Further interrogation using longitudinal study design and longer follow-up should continue to further confirm the lack of harm.

### Respiratory disease

Smoking is recognized as the most important cause of COPD [72] with a relative risk of dying of approximately 26 for men and 22 for women [66], and early quitting is associated with reduced morbidity and mortality[73]. Smoking has also been shown to increase the development of asthma, trigger asthma attacks and worsen outcomes of attacks [61, 74]. Other lung disorders that are causally linked with smoking include tuberculosis (TB) and idiopathic pulmonary fibrosis [61].

Cross-sectional designs are particularly problematic to investigate COPD as it usually takes several decades to develop [75] and because patients with COPD may be more likely to use quit aids such as e-cigarettes.

Despite mixed findings, studies judged to be of rigorous design (accounting for temporality, and former and current smoking) suggest that switching from cigarettes to e-cigarettes results in a reduction in exacerbations of COPD, with no evidence of long-term deterioration in lung function. The best evidence found no increased risk of asthma in ENDS users who were never smokers. There is a suggestion of short-term respiratory function changes in asthmatics using ENDS, but no evidence that it would translate to long-term impact.

### Cancer

Smoking-related cancers have been extensively studied [76] and include the mouth, throat, nose, sinuses, oesophagus, bladder, kidney, ureter, pancreas, stomach, liver, cervix and ovary, bowel and acute myeloid leukaemia [77].

Only two studies on cancer were identified and the association of e-cigarettes in the causation of cancer has not been explored in clinical studies to any extent, which may in part be due to the lack of a plausible biological pathway.

### Oral health

Oral cancer is the eleventh most common cancer worldwide [78], but oral health issues for ENDS have not been adequately studied.

### Mental health

Particular aspects in mental health patients include high prevalence of both smoking and ENDS use [79–81], preliminary evidence that ENDS are highly effective for smoking cessation in this group [80], and that this group is more prone to addiction [82, 83] and struggle to quit nicotine in the longer term [79]. Furthermore, nicotine itself may have an impact on symptoms and progression of the mental health condition [84].

Seven studies investigating mental health outcomes were identified in this review, but there were others reporting on mental health disease as a predictor of ENDS use [85, 86]. Interventional and longitudinal study designs are critical due to the bi-directional link between mental health disorders and ENDS use. Of two longitudinal studies in our review, one showed no deterioration in depressive symptoms and the other showed some deterioration, so no conclusion can be reached. Further studies are urgently required that are interventional in design and to investigate other health outcomes of switching from cigarettes to ENDS in this patient group.

### Informing policy

The findings of our review have implications for policy makers. Our review found that very few studies were sufficiently rigorous to form conclusions on health risks and were not rigorous enough to inform policy on tobacco harm reduction.

The European Commission published recently stated strong weight of evidence for risks of long-term systemic effects for CVD, respiratory cancers and poisoning and injuries; moderate for respiratory tract irritative damage and that other long-term adverse health effects, such as pulmonary disease, central nervous system and repro-toxic effects, cannot be established due to lack of consistent data [87]. Current European policies requires packaging for ENDS products to report the same information on toxicity and addictiveness as for cigarettes and tobacco products [88]. The findings of our review do not support these conclusions and should form part of the scientific basis for such policies.

Several of the studies included in this review that were neither high level of evidence nor of ‘good’ quality have nevertheless been influential in determining health policy. One such study [59] found that current e-cigarette users were twice as likely as never users to have had a MI. However, a subsequent re-analysis of the data [89] found that the majority of the MI outomes had preceded, on average by a decade, the first use of e-cigarettes [90]. Despite being retracted by the publishing journal [91], the article had already been widely disseminated [92] and cited [93], with potential lasting impacts on the perception of CVD health risks from the use of e-cigarettes.

Another invalid health scare informing policy from the use of ENDS occurred in 2019 with the “EVALI” outbreak which was initially widely reported as an outbreak of lipoid pneumonia due to vaping of nicotine [94]. It was soon recognised and reported as being due to vaping of black-market cannabinoid (THC) oils rather than vaping of nicotine, with the CDC in the US recommending that adults using nicotine-containing e-cigarette or vaping products as an alternative to cigarettes should continue and not go back to smoking [94].

The general public’s perception of health risks from ENDS does not reflect the available evidence and has become more negative according to the findings from two large surveys [95], whose authors underscored the urgent need to accurately communicate the risks of e-cigarettes to the public, which should clearly differentiate the absolute from the relative (to smoking) harms of e-cigarettes.

### Strengths and Limitations

We considered a MA of studies included in our review to be inappropriate, partly due to the common methodological flaws highlighted above and the vast heterogeneity between studies, for example in the definitions used for the exposure variable of ENDS use, and with regards to accounting for dual use, former use, duration and quantity of use.

We sought to identify only those articles where the main research question was on disease end-points from use of ENDS (not including heat-not-burn devices). The key disease end-points under investigation were mortality, CVD, respiratory and cancer as these make up the major health concerns from ENDS. We also searched for general health outcomes to identify the breadth of health outcomes being reported. There may be other research studies where health outcome was a secondary research question or fell outside of our search terms which may not have been captured in our study.

We were unable to study the differential impact from various types of ENDS products and different constituent compounds (e.g., in nicotine fluid). In addition, different types of ENDS have different levels of nicotine delivery and addictive properties, which are likely to change the harmful effects (from components other than nicotine) of any product due to type of use (e.g. magnitude, time, etc.).

The individual studies synthesized for our review may also have been included in meta-analyses that were included. This should be acknowledged, however, as we did not ourselves conduct pooling of results, we do not regard this as problematic.

Finally, the search strategy results were limited to English language reports, and there is a risk that potentially relevant studies reporting health outcomes with ENDS use were subsequently not included.

## Conclusion

To determine the net health impact of ENDs, the benefits of quitting smoking must be weighed against any harms (or benefits) from the use of ENDS. The wider impacts from the use of ENDS on society, such as new uptake in never smokers and nicotine addiction, must also be factored in, which were outside of the scope of this review.

Our review suggests the majority of studies on the use of ENDS products reported on negative health impacts with few reporting on health outcomes from switching from cigarettes to e-cigarettes. Future studies will need to prioritise an exploration of both potential harms and benefits. The strength of evidence and quality of the published studies is generally poor, yet some of these studies have been used to inform policy and are likely to have influenced public perception of health risks from the use of ENDS.

Our review has not demonstrated evidence that ENDS use is causative for any harmful CVD outcomes, and to the contrary, may be beneficial for hypertensive patients. Switching from cigarettes to e-cigarettes resulted in reduced exacerbations of COPD, with no evidence of long-term respiratory harm or deterioration in lung function. There was a suggestion from one study of short-term reductions in respiratory function in asthmatics, but no increased risk of asthma in ENDS users has been shown. Other health outcomes such as mental health, cancer and mortality have not been adequately studied to form a consensus on the health impact from ENDS use. However, the findings of our review did not negate the consensus held by many that ENDS use is safer than the risks posed from smoking cigarettes.

Overall, our review found very few studies were sufficiently rigorous to form conclusions on health risks. The research on ENDS use is not yet adequate to provide quantitative estimates about health risks. Consequently, the current body of evidence is inadequate for informing policy around tobacco harm reduction.

## Appendix

Table 5.

## Abbreviations

ADHD: Attention-deficit hyperactivity disorder
BMI: Body mass index
CAT: COPD assessment test
CAD: Coronary artery disease
CHD: Coronary heart disease
COPD: Chronic obstructive pulmonary disease
CVD: Cardiovascular disease
DBP: Diastolic blood pressure
ENDS: Electronic nicotine delivery systems
EC: Electronic cigarette
HR: Heart rate
MA: Meta-analysis
MI: Myocardial infarction
NASEM: National Academies of Sciences, Engineering and Medicine
NIH: National Institutes for Health
PAD: Peripheral arterial disease
PATH: Population Assessment of Tobacco Health
PTSD: Post-traumatic stress disorder
RCT: Randomized controlled trial
SBP: Systolic blood pressure
TB: Tuberculosis
THR: Tobacco harm reduction
